# Importance of Toxicokinetics to Assess the Utility of Zebrafish Larvae as Model for Psychoactive Drug Screening Using Meta-Chlorophenylpiperazine (mCPP) as Example

**DOI:** 10.3389/fphar.2018.00414

**Published:** 2018-04-26

**Authors:** Krishna Tulasi Kirla, Ksenia J. Groh, Michael Poetzsch, Rakesh Kumar Banote, Julita Stadnicka-Michalak, Rik I. L. Eggen, Kristin Schirmer, Thomas Kraemer

**Affiliations:** ^1^Department of Forensic Pharmacology and Toxicology, Zurich Institute of Forensic Medicine, University of Zurich, Zurich, Switzerland; ^2^Department of Environmental Toxicology, Swiss Federal Institute of Aquatic Science and Technology, Eawag, Dübendorf, Switzerland; ^3^Food Packaging Forum Foundation, Zurich, Switzerland; ^4^Department of Psychiatry and Neurochemistry, Institute of Neuroscience and Physiology, Sahlgrenska Academy, University of Gothenburg, Gothenburg, Sweden; ^5^Civil and Environmental Engineering, School of Architecture, EPFL, Lausanne, Switzerland; ^6^Institute of Biogeochemistry and Pollutant Dynamics, ETH Zurich, Zurich, Switzerland

**Keywords:** meta-chlorophenylpiperazine (mCPP), cocaine, psychoactive drugs, toxicokinetics, toxicity, behavior, melanin

## Abstract

The number of new psychoactive substances (NPS) increases rapidly, harming society and fuelling the need for alternative testing strategies. These should allow the ever-increasing number of drugs to be tested more effectively for their toxicity and psychoactive effects. One proposed strategy is to complement rodent models with zebrafish (*Danio rerio*) larvae. Yet, our understanding of the toxicokinetics in this model, owing to the waterborne drug exposure and the distinct physiology of the fish, is incomplete. We here explore the toxicokinetics and behavioral effects of an NPS, meta-chlorophenylpiperazine (mCPP), in zebrafish larvae. Uptake kinetics of mCPP, supported by toxicokinetic modeling, strongly suggested the existence of active transport processes. Internal distribution showed a dominant accumulation in the eye, implying that in zebrafish, like in mammals, melanin could serve as a binding site for basic drugs. We confirmed this by demonstrating significantly lower drug accumulation in two types of hypo-pigmented fish. Comparison of the elimination kinetics between mCPP and previously characterized cocaine demonstrated that drug affinities to melanin in zebrafish vary depending on the structure of the test compound. As expected from mCPP-elicited responses in rodents and humans, zebrafish larvae displayed hypoactive behavior. However, significant differences were seen between zebrafish and rodents with regard to the concentration-dependency of the behavioral response and the comparability of tissue levels, corroborating the need to consider the organism-internal distribution of the chemical to allow appropriate dose modeling while evaluating effects and concordance between zebrafish and mammals. Our results highlight commonalities and differences of mammalian versus the fish model in need of further exploration.

## Introduction

The production and consumption of synthetic drugs of abuse, also known as NPSs, have been increasing rapidly (Goosdeel, 2016), intensifying the need for an efficient assessment of their toxicity and behavioral effects. Zebrafish *(Danio rerio)* larvae have been suggested as an alternative to rodent models because, opposite to rodents, drug testing can be achieved with high information content at medium to high throughput. Yet, understanding of the chemical fate in zebrafish larvae is far from complete (Rihel and Ghosh, 2014). In our previous study we found that, upon waterborne exposure, cocaine accumulation was three-fold higher in zebrafish larval eye than in the brain or trunk (Kirla et al., 2016). This finding sheds light on potential binding sites of chemicals in zebrafish larvae. Cocaine, being a basic drug, is known to bind to melanin in human and rat hair (Nakahara et al., 1992; Cone et al., 1993; Joseph et al., 1997). Since zebrafish larvae are highly pigmented, especially in the eyes, binding to melanin could be a very important mechanism for the accumulation of basic drugs in this species. We hypothesized that in zebrafish larvae melanin plays a role in binding basic drugs, with affinities potentially differing depending on the chemical structure, as has been shown with synthetic melanin (Baweja et al., 1977).

To investigate this hypothesis, we chose the NPS mCPP, also a basic drug with psychoactive properties (Arbo et al., 2012) but structurally different from cocaine in having a piperazine ring. mCPP is an N-substituted piperazine with a molecular weight of 196.68 g/mol, an octanol-water partition coefficient (logP) of 2.06 and a pKa of 8.87. This chemical met our two selection criteria: Firstly, it is known to bind to melanin, at least in mammalian hair (Gaillard et al., 2013), but with presumably lower affinity than cocaine. The latter characteristic has not yet been directly shown with mCPP, but it is known for fluphenazine, an anti-psychotic therapeutic drug with a piperazine ring (Baweja et al., 1977; Tardy et al., 2014). Secondly, mCPP is a widely used synthetic drug of abuse, and we were interested in assessing its toxicity and behavioral effects in zebrafish larvae in order to compare them with mammals. In rodents, mCPP was shown to lead to reduced locomotion (Klodzinska et al., 1989; Lucki et al., 1989), and in humans is known to reduce psychomotor activity (Murphy et al., 1989; Broocks et al., 1997; Sabbe et al., 2001). In mammals, mCPP, i.e., the active drug, is known to be eliminated by phase-I oxidation and phase-II glucuronidation and sulfation (Mayol et al., 1994; Rotzinger et al., 1998).

Thus, using mCPP as an example, we here explore and discuss the (a) importance of melanin as an internal binding site of basic drugs in zebrafish larvae and (b) the need to consider kinetics and tissue distribution of psychoactive drugs to allow appropriate tissue dose modeling for interpretation of toxicity and behavior and for assessment of concordance between zebrafish and mammals.

## Materials and Methods

### Animal Husbandry and Ethics

Zebrafish (*Danio rerio*) of OBI strain were maintained in a flow-through system (Müller & Pfleger, Rockenhausen, Germany) at 28°C under a 14-h light/10-h dark cycle according to published guidelines (Nuesslein-Volhard and Dahm, 2002). Fish were fed twice daily a diet of live-hatched brine shrimp (*Artemia nauplia*) and flake fish food (Tetramin, Switzerland). Breeding was carried out by group crosses. Eggs were collected in the morning and raised in an incubator at 28°C with the same light/dark cycle as adults in reconstituted water [294.0 mg/l CaCl2.2H2O, 123.2 mg/l MgSO4.7H2O, 64.74 mg/l NaHCO3 and 5.7 mg/l KCl; ISO 15088:2007(E), 2007] in Petri dishes of 50–60 embryos per dish. All experimental procedures were performed in accordance with the animal protection guidelines and the experiments with the larvae were approved by the Swiss Cantonal Veterinary Office.

### Zebrafish Embryo Toxicity Test (zFET)

The mCPP was purchased from Lipomed, Switzerland (>98.5% pure) with special permission from the Swiss Federal Office for Public Health to use controlled substances and treated according to the institutional safety procedures. In order to determine the toxic and non-toxic concentrations of mCPP, the zFET test was performed as recommended in the guidelines of the Fish Embryo Toxicity Test, OECD, Test Guideline 236. Briefly, zebrafish embryos at two to four cell-stages were distributed one embryo per well in a 24-well plastic microtiter plate (Huber, Switzerland) and exposed to mCPP (10 embryos/concentration). Exposure concentrations were chosen based on initial range-finding tests, and included 0.01, 0.05, 0.1, 0.5, 1, 2.5, 5, 50, and 100 μM. Final exposure concentrations were achieved by dissolving 1 mM stock solution of mCPP in an appropriate volume of reconstituted water medium. Test solutions were exchanged with freshly prepared solutions daily. The embryos were monitored from 24 to 120 hpf and sub-lethal and lethal outcomes were noted in μM. Lethal and sub-lethal concentrations were calculated using the four-parameter sigmoidal dose-response (variable slope) model in GraphPad Prism^®^ 6.

### Toxicokinetics of mCPP

#### Uptake, Biotransformation and Elimination of mCPP

All exposures for toxicokinetic analyses were done with 5 dpf larvae in a 48-well plastic plate (Huber, Switzerland) at 28°C. For the uptake analysis, 16 larvae were incubated, one larvae per well, in 5 μM mCPP and were collected after 0.25, 1, 3, 6, 8, and 10 h exposure into pre-weighed 2 ml lysing matrix tubes with metal beads (MP Biomedicals, France). Samples were washed with phosphate buffered saline, snap frozen in liquid nitrogen and stored at -80°C until analysis. For elimination experiments, following 8 h of uptake, larvae were transferred into reconstituted water (drug-free medium) and sampled at 1, 3, 6, 18, 24, 27, and 48 h of depuration. As the lysing matrix tubes contained beads, making it difficult to aspirate all liquid medium, wet weights of 5–7 dpf larvae were instead measured in a separate experiment using 1.5 ml Eppendorf tubes, where liquid medium was gently aspirated before weighing. Larvae measured over 5–7 dpf gave an average weight of 360 ± 16 μg and therefore, 360 μg was used to calculate internal body concentrations (Supplementary Table S2A).

To analyze the kinetics of uptake and elimination, frozen samples were defrosted first at 4°C for 1 h and then at room temperature for about 2 h. Samples were homogenized by adding ammonium formate buffer (500 μL, 5 μM, pH 3.1) and using a Fast prep^®^- 24 Instrument (MP, Biomedicals, France). To the homogenate, internal standard (mCPP-D_3_) was spiked at a concentration of 5 μM. Extraction was carried out with 500 μL acetonitrile (Sigma Aldrich, Switzerland) and samples were centrifuged at 10,000 rpm for 5 min. Supernatant was collected into a fresh tube, mixed with eluent buffer (10 mM ammonium formate with 0.1% formic acid and acetonitrile with 0.1 % formic acid) and were injected into the LC-MS/MS (Applied Biosystems 5500 triple QTRAP, AB Sciex, Germany) for the analysis of mCPP and metabolites. MRM transitions were acquired for mCPP (197 → 154.1), OH-mCPP (213 → 134) and OH-mCPP glucuronide (389 → 213).

#### Distribution of mCPP in Zebrafish Larvae

##### Qualitative analysis by MALDI MSI

Spatial distribution of mCPP in zebrafish larvae was studied using MALDI MSI according to the protocol described in Kirla et al. (2016). Briefly, larvae were exposed to 5 μM mCPP in a similar way as for the uptake analysis. After 8 h of uptake, larvae were euthanized and frozen in Optimal Cutting Temperature media (Thermo Scientific^TM^, United States) on dry ice. Individual blocks were mounted onto the cryotome (Microm HM 560, Thermo Scientific^TM^, United States) and 16 μm-thick coronal, sagittal and transverse sections were made. Sections were collected on the indium tin oxide coated glass slides and immediately dried under vacuum to avoid any redistribution of the drug. MALDI matrix (10 mg/ml of 2,5-dihydroxybenzoic acid; 0.1% TFA/ACN 1:1) was applied on the samples and images were acquired on a Flashquant Workstation (AB Sciex, Germany) in positive ion mode. Images were processed using Tissue view software (AB Sciex, v1.1) and overlaid with the optical images.

##### Quantitative analysis by LC-MS/MS

To quantitate mCPP in different tissues, larval brain, eyes and trunk were dissected out as described in (Turner et al., 2014). Brains, eyes and trunks from 16 fish were pooled into different tubes and analyzed using LC-MS/MS with the method described in (Kirla et al., 2016). Wet weights of the dissected brain, eyes and trunk were measured individually before freezing (Supplementary Table S2B).

### Hypo-Pigmented Fish Experiments

Albinos (*slc45a2*) were obtained from the facility of Stephan Neuhauss from the University of Zurich and were raised in our facility as described above for the WT zebrafish. To produce hypo-pigmented fish chemically, zebrafish embryos were exposed to 200 μm of PTU by dissolving in appropriate volume of embryo medium at 22 hpf when melanin synthesis begins (Kimmel et al., 1995). Solutions were exchanged every day until 5 dpf. Albinos and PTU-treated larvae of 5 dpf were exposed to 5 μM mCPP, and its concentration after 8 h of uptake was measured in the whole-body homogenates and in the dissected tissues by LC-MS/MS as described above.

### Toxicokinetic Modeling

Toxicokinetic modeling was performed as described in Kirla et al. (2016). Briefly, one-compartment and multi-compartment (i.e., two compartment) models were applied to determine empirical rates of uptake and elimination for mCPP. The one-compartment concept (fitted one-compartment model 1) can be described by the following equation (Stadnicka et al., 2012):

$$\frac{d}{dt}C_{\mathrm{int}}(t)=k_{in}\cdot C_{w}(t)-k_{out}\cdot C_{\mathrm{int}}\left(t\right)$$

where C_int_(t) is the internal chemical concentration (mg kg^-1^), C_w_(t) is the chemical concentration in the water (mg L^-1^), k_in_ is the uptake rate constant (L kg^-1^h^-1^)and k_out_ is the elimination rate constant (h^-1^). Fitted one-compartment model 1 and measured internal concentrations of mCPP were compared with predicted values obtained by applying a simple one-compartment toxicokinetic model which assumes uptake and elimination of organic chemicals as a function of the octanol-water partition coefficient, as well as the lipid content, weight and trophic level of the species (Hendriks et al., 2001) (predicted one-compartment model 2). This model can also be described by the equation 1; however, here the uptake and elimination rate constants were not fitted but determined based on the physico-chemical properties of mCPP and larvae characteristics as described in Hendriks et al. (2001). All the parameters and equations used for the determination of uptake and elimination rate constants in predicted one-compartment model 2 are presented in the Supplementary Table S1 (equations S1–S5).

A multi-compartment model (fitted multi-compartment model 3) was applied as described in detail in (Kirla et al., 2016), in order to account for the mCPP distribution in the larvae. In this model, eyes are distinguished as a separate compartment due to uptake and elimination processes different than for the rest of the body. Therefore, different rate constants can be determined for the eyes and for the rest of the body:

$$\frac{d}{dt}C_{\mathrm{int}\_eyes}(t)=k_{\mathrm{in}\_eyes}\cdot C_{w}(t)-k_{out\_eyes}\cdot C_{\mathrm{int}\_eyes}\left(t\right)$$

$$\frac{d}{dt}C_{\mathrm{int}\_rest}(t)=k_{\mathrm{in}\_rest}\cdot C_{w}(t)-k_{out\_rest}\cdot C_{\mathrm{int}\_rest}\left(t\right)$$

where C_int_eyes_(t) is the chemical concentration in the larvae’s eyes (mg kg^-1^), C_int_rest_(t) is the chemical concentration in the larvae’s tissues and organs other than eyes (mg kg^-1^), C_w_(t) is the chemical concentration in the water (mg L^-1^); k_in_eyes_ and k_in_rest_ are the uptake rate constants (L kg^-1^h^-1^) and k_out_eyes_ and k_out_rest_ are the elimination rate constants (h^-1^) in the eyes and in the rest of the body, respectively.

The relationship between chemical concentrations in eyes and in the whole fish was determined over time by the following equation:

$$C_{\mathrm{int}\_larvae}(t)=\frac{C_{\mathrm{int}\_eyes}(t)\cdot W_{eyes}+C_{\mathrm{int}\_rest}(t)\cdot W_{rest}}{W_{eyes}+W_{rest}}$$

where C_int_larvae_(t) is the chemical concentration in the whole larvae (mg kg^-1^), W_eyes_ is the weight of eyes (kg) and W_rest_ is the weight of larvae’s tissues and organs other than eyes (kg). Larvae’s weight is the sum of W_eyes_ and W_rest_.

All models were implemented and solved using ModelMaker (version 4.0, Cherwell Scientific Ltd., Oxford, United Kingdom) with the settings described in the Supporting Information (SI – page 9).

### Monitoring of Locomotory Behavior

Behavioral testing was performed on 5 dpf larvae. Larvae were distributed one per well in a 48-well plate with embryo medium (500 μL) and incubated for 3–4 h for acclimatization in the housing incubator. After acclimatization, larvae were exposed according to one of the two different mCPP concentration setups: setup 1, low concentrations – 0.01, 0.05, 0.25, 0.5, and 1 nM and setup 2, high concentrations – 0.001, 0.01, 1, 2.5, and 5 μM. Untreated larvae served as controls in each of the experiments. Drug challenge was conducted on three different plates on independent days with *n* = 8 larvae/concentration or untreated controls/plate. Movement of each larva was monitored using the ZebraBox^TM^ video tracking system (View Point Life Sciences, Version 3, Lyon, France) with a camera frame rate of 25 frames per second. In the software user interface, distance traveled was categorized into three types of movements: low speed movements (<2 mm/s), medium speed movements (2–10 mm/s) and high speed movements (>10 mm/s). The average distance traveled was then calculated from the different speed movements.

Following testing behavior on instant drug exposure, each plate was immediately placed on the recording platform and each individual animal was tracked for 70 min with alternating dark and light phases every 10 min. For recording behavior after extended drug exposure, a different set of fish were exposed to mCPP in another plate and placed in the incubator for 8 h before measuring the locomotor activity using the same protocol as for immediate drug exposure. Data was exported to and analyzed in Microsoft Excel 2010 and the graphs were plotted in GraphPad Prism^®^ (Version 6 for Windows, CA, United States).

### Statistical Analysis

All graphs were plotted using GraphPad Prism^®^ (Version 6). Statistical analysis for the behavior data was performed using RStudio (Version 0.98.1103) by repeated measures Analysis Of Variance (ANOVA) as described in Kirla et al. (2016). Briefly, data were analyzed in three steps: first, data were assessed for the significance of independent factors, i.e., time, concentration and plate with the dependent factor being the locomotor activity. Next, data were segregated based on the light and dark conditions (considering from the first light condition that followed dark period) and ANOVA was performed. Finally, at each lighting condition, ANOVA was performed comparing each concentration to control to test if there is a significant effect followed by Bonferroni’s *post hoc* analysis. Statistical significance was set at α = 0.003.

## Results

### mCPP Uptake, Biotransformation and Elimination in Zebrafish Larvae

**FIGURE 1:**
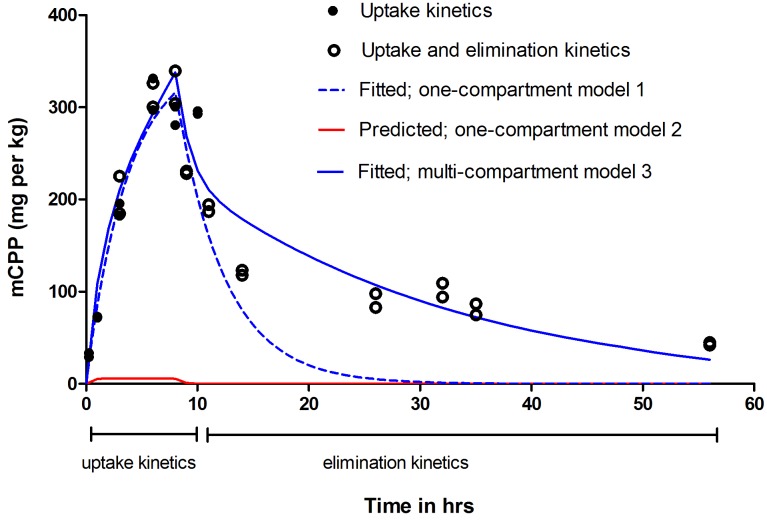
Uptake and elimination kinetics of mCPP in zebrafish larvae. Larvae (16 per treatment) at 5 dpf were exposed to 5 μM mCPP for 8 h and thereafter placed in clean medium for up to 48 h. A set of untreated larvae served as control. At different time points during the uptake and elimination phase, mCPP concentrations in the whole-body homogenates were measured by LC-MS/MS. Filled circles show data from two independent experimental replicates focusing on uptake kinetics; open circles show data from two additional independent experimental replicates focusing on both uptake and elimination kinetics. Dashed line in blue shows the fitted one-compartment model 1; solid line in red shows the prediction of uptake and elimination based on one-compartment toxicokinetic model 2; solid line in blue shows the fitted multi-compartment toxicokinetic model 3.

To quantify the kinetics of mCPP uptake, zebrafish larvae were exposed to 5 μM (0.98 mg/L) mCPP and the internal concentrations were quantified over time. The 5 μM mCPP concentration was the highest one not affecting the larvae phenotype when exposed at 4 dpf for 24 h (Supplementary Figure S1A). mCPP was detected in the whole-body homogenates already at the earliest assessed time point (15 min) and the uptake increased gradually, reaching measured internal concentrations of 308 ± 18 mg/kg within the 8 h of exposure (**Figure 1**). Based on the fitted one-compartment model 1 (see Equation 1: Materials and Methods – Toxicokinetic modeling), the uptake rate constant, k_in_, was determined to be 112.9 ± 7.4 L kg^-1^h^-1^.

To follow the elimination of mCPP over time, zebrafish larvae were transferred into clean water after 8 h of uptake. Based on the measured data, the half-lives could be distinguished into two values: an initial half-life of 4.5 h during the first 6 h of the elimination phase followed by a longer half-life of 28.5 h during the rest of the duration (**Figure 1** and Supplementary Figure S2). About 13% (i.e., 40 mg/kg) remained after 48 h of elimination with an overall elimination rate constant, k_out_, of 0.25 ± 0.03 h^-1^ as derived from the fitted one-compartment model.

Zebrafish larvae biotransformed mCPP by oxidation to OH-mCPP, followed by conjugation to a glucuronide (Supplementary Table S3). About 1–2% of the parent compound taken up underwent oxidation and glucuronidation over the same time span as analyzed for the uptake kinetics, i.e., 8 h.

The predicted one-compartment toxicokinetic model (Materials and Methods – Toxicokinetic modeling, model 2) (Kirla et al., 2016), which assumes passive uptake and elimination of mCPP based on its octanol-water partition coefficient (log *P* = 2.06), predicted much lower whole-body concentrations than those actually measured (**Figure 1**). Correcting the logP value based on the pKa and pH (log *D* = 0.91), would result in the predicted internal concentrations much lower than those presented in **Figure 1**.

### mCPP Binding in Zebrafish Eye Is Reversible

To visualize the distribution pattern of mCPP in the larvae of zebrafish after exposure to 5 μM mCPP for 8 h, we used MALDI-MSI to analyze coronal, sagittal and transverse sections. Coronal sections revealed mCPP accumulation with high intensity in the head region and with lesser intensity in the trunk (Supplementary Figures S3A,B). Sagittal sections showed mCPP signal in the brain and eye (Supplementary Figures S3C,D). Transverse sections made through forebrain, midbrain and hindbrain confirmed the presence of mCPP in the brain (Supplementary Figures S3E–J). Sagittal sections of unexposed control fish showed no mCPP signal (Supplementary Figures S3K,L).

**FIGURE 2:**
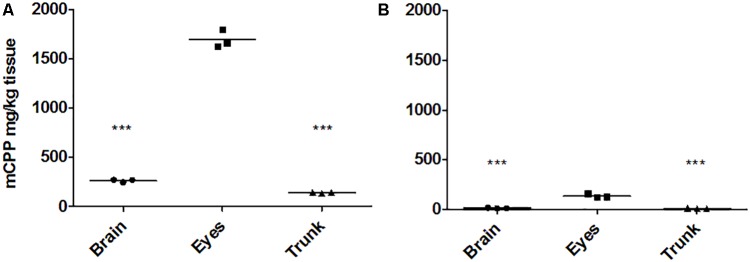
mCPP concentrations in dissected tissues quantified by LC-MS/MS. Zebrafish larval brain, eyes, and trunk were dissected from 16 larvae after being exposed to 5 μM mCPP or unexposed as controls either for 8 h **(A)** or for 8 h followed by 48 h of elimination **(B)** and analyzed for mCPP. Values are reported as original data with each data point representing the information obtained from a pool of 16 larval tissues in one of three independent experiments. Horizontal lines represent the mean of the experimental replicates. One-way ANOVA followed by Tukey’s multiple comparison test revealed a significant difference for brain versus eyes and trunk versus eyes (^∗∗∗^*p* < 0.0001) for both sampling time points **(A,B)**.

To quantify the distribution of mCPP in the larvae after an exposure for 8 h to 5 μM mCPP, brain, eyes, and trunk were dissected and mCPP was quantified by LC-MS/MS. The by far highest concentration of mCPP was found in the eyes (1694 ± 76 mg/kg) followed by the brain (263 ± 11 mg/kg) and the trunk (143 ± 5 mg/kg) (**Figure 2A**).

Due to the high accumulation of mCPP in the eyes, one-compartment approaches could not describe the data well (**Figure 1**). Therefore, a multi-compartment toxicokinetic model was applied as described in (Kirla et al., 2016) in order to distinguish eye as a separate compartment from the rest of the body (Supplementary Figure S4). Modeling results demonstrate that, while the distribution in the brain and trunk is about 20-fold higher than predicted by the simple partitioning assumption, the distribution in the eye compartment is over 250-fold higher than predicted. The k_in_ derived from the multi-compartment model in the eyes (i.e., k_in__eyes, Equation 2) was 325 ± 5.9 L kg^-1^ h^-1^ and 143.2 ± 50 L kg^-1^ h^-1^ in the rest of the body (i.e., k_in__rest, Equation 3).

Measuring the mCPP levels in the dissected brain, trunk and eyes after 48 h in clean water revealed significant elimination from all tissues examined. Of the initial concentrations accumulated in the different tissues after 8 h of uptake, 4.5% (11.9 ± 3.9 mg/kg) remained in the brain, 5% (7.3 ± 2.6 mg/kg) in the trunk and 8% (134.5 ± 16 mg/kg) in the eyes, with the latter compartment still showing significantly higher mCPP concentrations compared to the two former ones (**Figure 2B**). Here, the k_out_ in the rest of the body excluding eyes was 0.8 ± 0.3 h^-1^, while it was much slower in the eyes with a rate constant of 0.05 ± 0.003 h^-1^. The fitted multi-compartment model gave half-lives of 13 h and 48 min for the elimination from the eyes and the rest of the body, respectively.

### Hypo-Pigmented Fish Accumulate Much Less mCPP Than Wild-Type Fish

**FIGURE 3:**
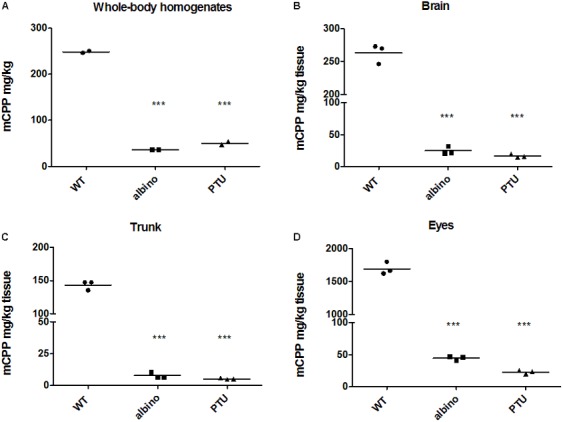
mCPP concentrations quantified by LC-MS/MS in the whole-body homogenates and dissected tissues of wild type and hypo-pigmented fish. Wild-type, albino and PTU-treated fish at 5 dpf (16 larvae per treatment) were either exposed to 5 μM mCPP or unexposed as controls for 8 h and mCPP was quantified in larval whole-body homogenates **(A)** or in dissected brain **(B)**, trunk **(C)**, and eyes **(D)**. Values are reported as original data with each data point representing the information obtained from a pool of 16 larval tissues in one of the two or three independent experiments. Horizontal lines represent the mean of the experimental replicates. One-way ANOVA followed by Tukey’s multiple comparison test revealed significant difference between albino versus wild-type and PTU versus wild-type fish both for whole-body homogenates and all dissected tissues (^∗∗∗^*p* < 0.0001).

To test our hypothesis of melanin serving as a binding site involved in drug accumulation, we used two types of hypo-pigmented fish: albinos (*slc45a2*) (Tsetskhladze et al., 2012) and PTU treated fish. For the latter, zebrafish embryos at 22 hpf were exposed to 200 μM PTU, a standard treatment used to prevent pigmentation of the embryos (Karlsson et al., 2001; R, 2002). mCPP levels in the whole-body homogenates, as well as in dissected brain, trunk and eyes, were compared between the WT, albinos and PTU-treated fish after 8 h of exposure to 5 μM mCPP. Whole-body concentrations of mCPP were about 7 times lower in the albinos (36 mg/kg) and 5 times lower in the PTU-treated fish (49 mg/kg) compared with the WT larvae (250 mg/kg) (**Figure 3A**). mCPP concentrations in the dissected tissues from hypo-pigmented fish were about 10 times lower in the brain (**Figure 3B**), 20 times lower in the trunk (**Figure 3C**) and 50 times lower in the eyes (**Figure 3D**) compared to the concentrations in the tissues of WT fish.

### mCPP Causes Hypoactivity in Zebrafish Larvae

**FIGURE 4:**
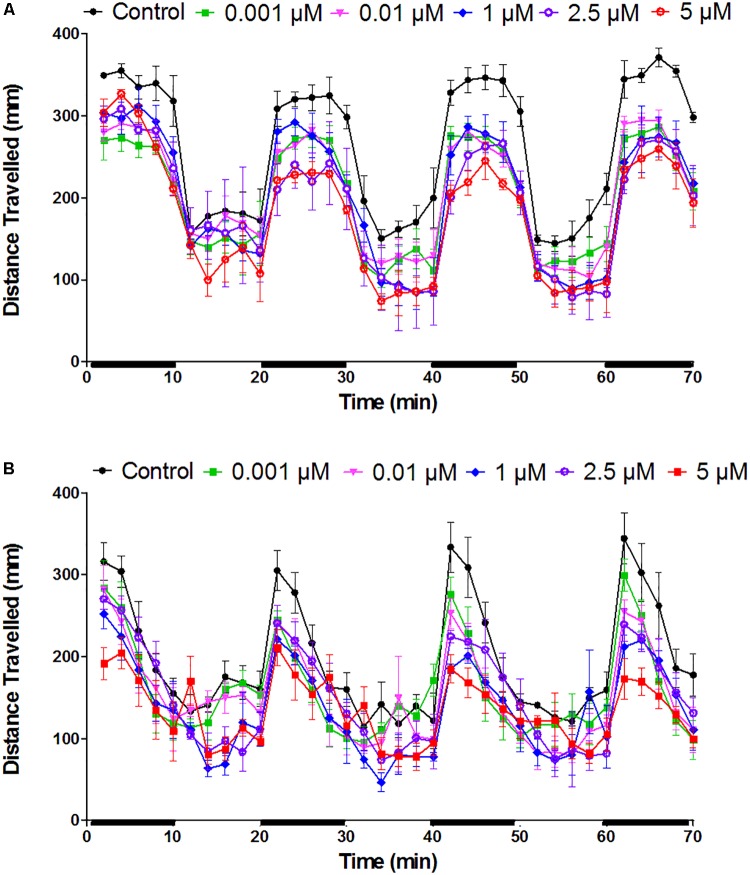
Effect of mCPP on the locomotor activity of zebrafish larvae. Larvae of 5 dpf were exposed to different concentrations of mCPP and tracked either immediately **(A)** or after 8 h of exposure **(B)**. Distance traveled by the fish was analyzed every 2 min for 70 min with dark (black bars on X-axis) and light phases alternating every 10 min. Data were assessed using repeated measures ANOVA. Values are reported as mean ± SEM from 3 independent experiments (*n* = 8 larvae/concentration or control/experiment).

We determined the effects of mCPP on the locomotory behavior of 5 dpf WT zebrafish larvae. Zebrafish larvae were exposed to different concentrations of mCPP and locomotor activity was measured either immediately after the exposure start (**Figure 4A**) or after 8 h of exposure (**Figure 4B**). At both time points, locomotor activity of mCPP-exposed larvae was lower compared to the untreated (mCPP-free) controls (**Figure 4**, Supplementary Table S4, and Supplementary Figures S5, S6), with the effect being significant at concentrations ≥ 0.001 μM. However, there was no apparent concentration-dependency in the hypoactive behavior.

## Discussion

This study aimed to advance knowledge on the suitability of zebrafish larvae as a vertebrate model to rapidly evaluate the toxicokinetics, toxicity and behavioral effects of classical and NPSs. As previously observed for cocaine (Kirla et al., 2016), mCPP is rapidly taken up by the larvae and dominantly accumulates in the eye. Using hypo-pigmented fish, we demonstrate that the accumulation in the eye compartment is mediated by melanin. Zebrafish larvae respond to mCPP with reduced activity as rodents and humans; however, whether the mechanisms leading to this behavior are similar requires further investigation. Significant differences were seen between zebrafish and rodents with regard to the concentration-dependency of the behavioral response and the comparability of tissue levels, corroborating the need to consider tissue-specific chemical distribution to allow appropriate dose modeling while evaluating effects and concordance between zebrafish and mammals. Our results thus highlight commonalities and differences of mammalian versus the fish model that need further exploration.

### Melanin Is an Important Binding Site for Basic Drugs in Zebrafish Larvae

**FIGURE 5:**
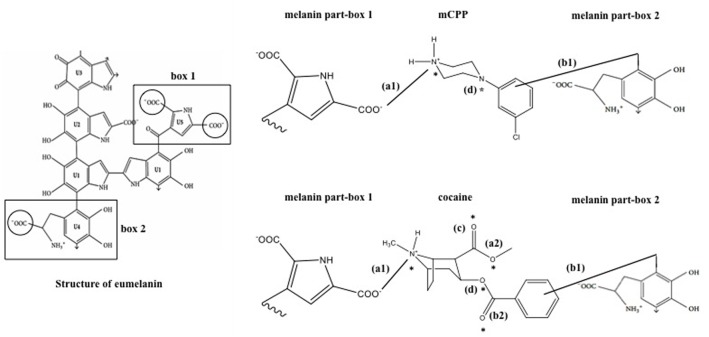
Schematic representation of possible binding sites of melanin with mCPP and cocaine. Box 1 and box 2 are selected sites of melanin proposed to be important for the interactions with chemicals. (a1) an example of an ionic interaction between a carboxylic acid and a protonated nitrogen (solid line) between melanin and cocaine/mCPP; (a2) additional ionic interaction site in cocaine with a carboxylic acid group; (b1) interactions between aromatic rings of melanin and cocaine/mCPP (solid line); (b2) an example of a double bond site in cocaine for the interaction with aromatic rings of melanin; (c) an example of a charge-transfer reaction site (oxygen) possible only in cocaine; (d) an example of hydrogen bridging site with five hydrogen acceptor sites in cocaine and only two in mCPP (indicated by asterisk). For example, Eumelanin structure has been adapted and modified from Karlsson and Lindquist (2016).

Our study demonstrates that melanin acts as an important binding site for basic drugs in zebrafish larvae, with the eye being the dominant accumulation compartment. We previously hypothesized the role of melanin in cocaine binding (Kirla et al., 2016); here we provide experimental proof for this hypothesis by studying mCPP distribution in hypo-pigmented zebrafish larvae versus WT fish. In our previous study, we observed that cocaine accumulation in the eye played no role in the behavioral effects analyzed (Kirla et al., 2016). Therefore, it is unlikely that mCPP accumulation in the larval eye measured in this study played any role in the hypoactivity seen. We conducted another study to evaluate the effect of accumulated cocaine on the zebrafish larval eye by electroretinography, which revealed that cocaine accumulation in the retina does not adversely affect outer retina function. It leads, however, to increased light sensitivity, a finding requiring further exploration to explain the mechanisms underlying this effect (Niklaus et al., 2017). The role of pigmentation in drug accumulation has been originally demonstrated in rabbits and hamsters where phenothiazines accumulated in the eyes of WT but not of albino animals (Potts, 1962). Later, binding to synthetic melanin or to hair was also shown for other aromatic compounds, such as chloroquine and cocaine (Potts, 1964; Larsson and Tjalve, 1979; Borges et al., 2003). All these compounds are basic drugs. Three striking features of melanin are thought to contribute to its ability to bind basic drugs: first, its high number of carboxylic acid residues which, with their negative charge, can lead to ionic interactions with a protonated nitrogen in the binding drug [**Figure 5**-(a1)] (Larsson, 1993; Karlsson and Lindquist, 2016); second, its great number of aromatic rings, which can contribute to interactions with the pi bonds of the binding drug [**Figure 5**-(b1)] (Reilly et al., 2015); and third, its electron accepting and donating properties, which can be involved in charge-transfer reactions with an electron donating or accepting substance [**Figure 5**-(c)] (Mason, 1977). Other mechanisms, such as hydrogen bonding [**Figure 5**-(d)] and van der Waal’s interactions, can be suggested as a fourth type of interaction (Mason, 1977; Karlsson and Lindquist, 2013, 2016).

We here show that basic drug binding by melanin is a phenomenon occurring also in the zebrafish eye. Furthermore, we demonstrate that the strength of binding varies depending on the physico-chemical properties of the drug (Zane et al., 1990) and the type of bonding (Mason, 1977; Tjälve et al., 1981). Accumulation of mCPP in the eye is much more reversible than that of cocaine: after 48 h of elimination, only 8% of the maximum accumulated mCPP amount persisted in the eyes, compared to 87% for cocaine, as observed in our previous study (Kirla et al., 2016). Referring to the four mechanisms listed above, we suggest several important structural differences that may contribute to stronger interactions of melanin with cocaine compared to mCPP (**Figure 5**). First, cocaine has one more site available for ionic interaction between the carboxylic acid residues in cocaine [**Figure 5**-(a2)] and a protonated nitrogen in melanin. Then, while both cocaine and mCPP have an aromatic ring for pi interactions with melanin [**Figure 5**-(b1)], cocaine contains two more pi bonds than mCPP [**Figure 5**-(b2)]. Furthermore, cocaine, with its oxygen atoms [**Figure 5**-(c)], can interact with melanin via charge transfer (Powell et al., 2007), while for mCPP this mechanism does not seem possible. Cocaine, moreover, has three more opportunities for hydrogen bridging with the donor melanin, i.e., cocaine can accept five hydrogen bonds (represented by asterisk) (CID=446220 National Center for Biotechnology Information, 2017) while mCPP can accept only two [**Figure 5**-(d)] (CID=1355 National Center for Biotechnology Information, 2017). Taken together, these mechanisms plausibly explain why the binding of cocaine to melanin is stronger, resulting in cocaine’s much slower release from the eye compared to mCPP. Relating these structural properties to the affinity of binding to melanin should aid in the prediction of basic drug binding to melanin and retention in the zebrafish eye.

### mCPP Is More Acutely Toxic Than Cocaine

Cocaine and mCPP also differ in their uptake kinetics and toxicity. The uptake rate constant, k_in_, for mCPP (112.9 L kg^-1^ h^-1^) is 10 times higher than for cocaine (12.9 L kg^-1^ h^-1^) (Kirla et al., 2016). Because of the faster uptake, the 5 μM exposure concentration of mCPP resulted in internal whole-body and tissue concentrations almost equal to those occurring after exposure to 50 μM of cocaine. Based on comparing model predictions for passive uptake and experimental data, we already suggested active uptake mechanisms for cocaine (Kirla et al., 2016). For mCPP, they are apparently even more important. This difference cannot be explained by the simplicity of the applied model. While increasing the model complexity generally improves model performance (Rowland et al., 2017), and more sophisticated Physiologically Based Toxicokinetic (PBTK) models have already been developed for adult stages of zebrafish (Péry et al., 2014; Brinkmann et al., 2016), no approach has, as of yet, been proposed for fish larval stages, for which chemical internal distribution differs. It has, however, been shown that the one-compartment model applied here is in agreement with the PBTK models regarding the prediction of chemical concentrations in whole fish (Stadnicka et al., 2012). Finally, the difference in uptake of mCPP and cocaine is also corroborated when comparing measured (logBCF = 2.65) and predicted bioconcentration factors (logBCF = 0.925; regression based method by the US Environmental Protection Agency’s EPISuite program^TM^) (EPISuite^TM^; US EPA, 2017). For cocaine, it has been demonstrated that the uptake across the blood-brain barrier is carried out by a proton-antiporter (Chapy et al., 2014). To our knowledge, the type of active transporters for mCPP is not yet known. Based on a previous study, we can, however, propose a possible mechanism: OCTs, which have been shown to be involved in the active uptake of imatinib, another piperazine drug, in human cells (Thomas et al., 2004). The fact that mCPP is present as a cation under physiological pH supports this proposal. The expression and function of OCTs has been previously demonstrated in zebrafish in tissues such as heart, kidney, brain, eye and liver (Mihaljevic et al., 2016). Therefore, the higher uptake of mCPP compared to cocaine may be explained by the fact that the type of transporters involved for mCPP are more prominent. This proposal opens up avenues for future investigations on the molecular mechanisms of mCPP uptake.

Compared to cocaine (Kirla et al., 2016), mCPP showed higher toxicity in short-term 24 h exposure at 4 dpf (Supplementary Figure S1A). From this data, the INTC, i.e., the concentration at which no significant effects were seen for mCPP, was estimated to be almost 7 fold lower (2.25 mmol/kg) compared to cocaine (14.65 mmol/kg) (Supplementary Information – equation S6). To assess the developmental effects of mCPP, zebrafish embryos were exposed from 0-5 dpf (Supplementary Figure S1B). The internal lethal and sub-lethal concentrations were estimated and compared to the acute toxicity of cocaine (Supplementary Information – equation S7). The ILC_50_ of mCPP is six-fold lower (51.75 mmol/kg) than that for cocaine (316.7 mmol/kg) and IEC_50_ is 50 times lower (0.47 mmol/kg) than for cocaine (25.1 mmol/kg). The sub-lethal effects noted upon mCPP exposure, such as heart oedema and yolk sac oedema, were more severe compared to cocaine. Furthermore, other effects such as protruded mouth and bent body axis were observed only on exposure to mCPP. These results suggest that mCPP not only can elicit developmental effects, a mode of action that has never been investigated in mammals, but that, moreover, different mechanisms may be involved in the developmental toxicity of cocaine and mCPP.

### Toxicokinetics and Behavioral Effects of mCPP in Zebrafish Larvae: Evaluation of Concordance and Discordance to Mammals

Although zebrafish larvae and mammalian models differ in the exposure routes for chemicals, the kinetics and behavioral effects can be compared based on internal concentrations. When comparing the uptake rate constant, k_in_ (L kg^-1^ h^-1^), from the exposure medium into the larvae with the absorption rate constant, k_a_ (h^-1^), from plasma in humans, the k_in_ of mCPP in zebrafish larvae (112.9) was almost 40 times higher than the k_a_ in humans (2.79) upon oral administration (Feuchtl et al., 2004). One possible explanation for the high uptake rate in zebrafish larvae versus humans could be that, in terms of the barriers to drug absorption present in these species, the intestinal barrier in humans may be stronger than the skin barrier or the gills in zebrafish larvae. However, since gill lamellae in zebrafish larvae become completely functional only from 12 to 14 dpf, the major barrier at this stage would be skin (Rombough, 2002). The active transport mechanisms could further contribute to higher uptake in zebrafish larvae.

Biotransformation of mCPP in zebrafish larvae is concordant to oxidation (phase I) and glucuronidation (phase II) in mammals (Mayol et al., 1994; Rotzinger et al., 1998). mCPP biotransformation in rodents is known to also lead to phase II sulfation (Mayol et al., 1994), but no sulfate conjugates were observed in zebrafish larvae. Although the expression of several SULTs has been characterized at this developmental stage in zebrafish, the physiological relevance of their expression is not yet clear and needs further investigation (Yasuda et al., 2008; Mohammed et al., 2012). Thus, it is not yet known if the SULT responsible for the biotransformation of mCPP is active in 5 dpf zebrafish larvae.

The initial 4.5 h elimination half-life (*t*_1/2_) of mCPP in zebrafish larvae shows concordance to the t_1/2_ of 4 h in humans (Feuchtl et al., 2004), which is striking given the differences in the size and routes of elimination, i.e., whole-body in the larvae vs. plasma in humans. This could be explained by the fact that different half-lives were obtained in the eyes (*t*_1/2_ = 13 h) and the rest of the body (*t*_1/2_ = 48 min). Therefore, because of the accumulation of mCPP in the eyes, the multi-compartment model best fits the toxicokinetics of mCPP.

Zebrafish larvae, tested over a wide concentration-range (0.00001–5 μM mCPP), respond consistently with significant hypoactive behavior at 0.01 μM or above, but the behavioral effect does not show concentration-dependency. In rodents, mCPP also leads to decreased locomotor activity, but in a dose-dependent manner when administered via intraperitoneal or sub-cutaneous injection (Klodzinska et al., 1989; Lucki et al., 1989; Gleason et al., 2001). mCPP is known to interact with different serotonin (5-HT) receptors and modulate the release of serotonin. In rodents, the hypoactivity has been demonstrated to occur through activating 5-HT_C_ receptors (Klodzinska et al., 1989; Gleason et al., 2001). Zebrafish larvae have a serotonergic neurotransmission system highly similar to mammals (Brustein et al., 2003; McLean and Fetcho, 2004; Panula et al., 2006, 2010). Therefore, the hypoactive behavior observed in zebrafish larvae could also be due to alterations in the serotonin neurotransmission. A 5 μM mCPP (≈1.4 mg/kg external concentration) exposure to zebrafish larvae resulted in a brain concentration of 0.3 ng/μg after 8 h exposure. In rodents, a 10 times lower brain concentration (0.02 ng/μg) was achieved 1 h post-injecting 10 mg/kg mCPP (Fuller et al., 1981) which led to hypoactive behavior (Gleason et al., 2001). The reason for the higher brain concentrations in zebrafish larvae compared to rodents could be the incomplete development of the blood-brain barrier at this stage (Jeong et al., 2008). Yet, if we assume linear uptake of mCPP across the tested concentration range that resulted consistently in significant hypoactivity in zebrafish larvae (0.01–5 μM), the lowest predicted effective brain concentration would be 0.0006 ng/μg (resulting from 0.01 μM), which is far lower than the reported 0.02 ng/μg of mCPP in rodent brain showing significant hypoactive behavior. In rodents, chronic exposure of mCPP resulted in behavioral tolerance (Ulrichsen et al., 1992; Fone et al., 1998). Therefore, in zebrafish larvae, irrespective of the brain concentrations, a hypoactive behavioral tolerance might explain the lack of a concentration-dependent effect, although tolerance is generally expected upon chronic exposure conditions. Therefore, future research should address whether zebrafish are capable of developing tolerance also after much shorter exposure durations. Another potential explanation is that high accumulation of mCPP in the brain or eyes may have led to disturbances in the brain or visual signaling, contributing to hypoactive behavior.

## Conclusion

Our data shows that, similar to mammals, melanin acts as a binding site for accumulation of basic drugs also in zebrafish larvae. The affinity of chemicals to melanin differs depending on the chemicals’ structure, as seen for mCPP and cocaine. These differences in interactions with melanin contribute to the differences in uptake, binding and elimination of basic drugs from zebrafish larvae, thus influencing the toxicokinetics to a great extent. Given the high uptake of mCPP in zebrafish larvae, we suggest the presence of active transport mechanisms operating at this stage. Finally, zebrafish larval responses to mCPP are both concordant and discordant compared to mammals. Despite the differences in effective tissue levels in zebrafish larvae and rodents, we observed similar behavioral responses. While this may suggest that zebrafish larvae could be a useful model to study serotonin-targeting drugs, further investigations with other serotonin-targeting drugs are needed to support this proposal.

## Author Contributions

KK, KG, RE, KS, and TK designed the research. KK, MP, and RB performed the research. KK, KG, JS-M, KS, and TK analyzed the data. KK, KG, and KS wrote the paper.

## Conflict of Interest Statement

The authors declare that the research was conducted in the absence of any commercial or financial relationships that could be construed as a potential conflict of interest.

## Abbreviations

dpf: days post fertilization
hpf: hours post fertilization
IEC_50_: internal effective concentration
ILC_50_: internal lethal concentration
INTC: maximum internal non-toxic concentration
LC-MS/MS: liquid chromatography coupled to tandem mass spectrometry
MALDI MSI: matrix assisted laser desorption ionization mass spectrometry imaging
mCPP: meta-chlorophenylpiperazine
MNTC: maximum non-toxic concentration
MRM: multiple reaction monitoring
NPS: new psychoactive substances
OCTs: organic cation transporters
OECD: Organisation for Economic Co-operation and Development
PTU: phenylthiourea
SULTs: sulfotransferases
WT: wild-type
zFET test: zebrafish embryo toxicity test
