# Supplementary material for: Importance of Toxicokinetics to Assess the Utility of Zebrafish Larvae as Model for Psychoactive Drug Screening Using Meta-Chlorophenylpiperazine (mCPP) as Example
Source: Front Pharmacol. 2018 Apr 26;9:414. doi: 10.3389/fphar.2018.00414 (PMC5932571; doi:10.3389/fphar.2018.00414)
Supplement: Supplementary file 1 [file Presentation_1.PDF]

Supplementary information

Importance of toxicokinetics to assess the utility of zebrafish larvae as model for psychoactive drug screening using meta-chlorophenylpiperazine (mCPP) as example

Krishna Tulasi Kirla[1,2], Ksenia J. Groh[2,3], Michael Poetzsch[1], Rakesh Kumar Banote[4], Julita Stadnicka-Michalak[2,5], Rik I.L. Eggen[2,6], Kristin Schirmer[2,5,6]*, Thomas Kraemer[1]

[1] University of Zurich, Zurich Institute of Forensic Medicine, Department of Forensic Pharmacology and Toxicology, Zurich, 8057, Switzerland
[2] Eawag, Swiss Federal Institute of Aquatic Science and Technology, Department of Environmental Toxicology, Duebendorf, 8600, Switzerland
[3] Food Packaging Forum Foundation, Zurich, 8045, Switzerland
[4] University of Gothenburg, The Sahlgrenska Academy, Institute of Neuroscience and Physiology, Department of Psychiatry and Neurochemistry, S-41345 Gothenburg, Sweden
[5] EPFL, School of Architecture, Civil and Environmental Engineering, Lausanne, 1015, Switzerland
[6]ETHZ, Institute of Biogeochemistry and Pollutant Dynamics, Zurich, 8092, Switzerland

SUMMARY OF SI APPENDIX

Supporting Figures S1 – S6

Figure S1: page 2

Figure S2: page 3

Figure S3: page 4

Figure S4: page 5

Figure S5: page 6

Figure S6: page 7

Supporting Equations and Parameters: pages 8-10

Supporting Tables S1 – S4

Table S1: page 8

Table S2: page 11

Table S3: page 12

Table S4: page 13

References: page 14

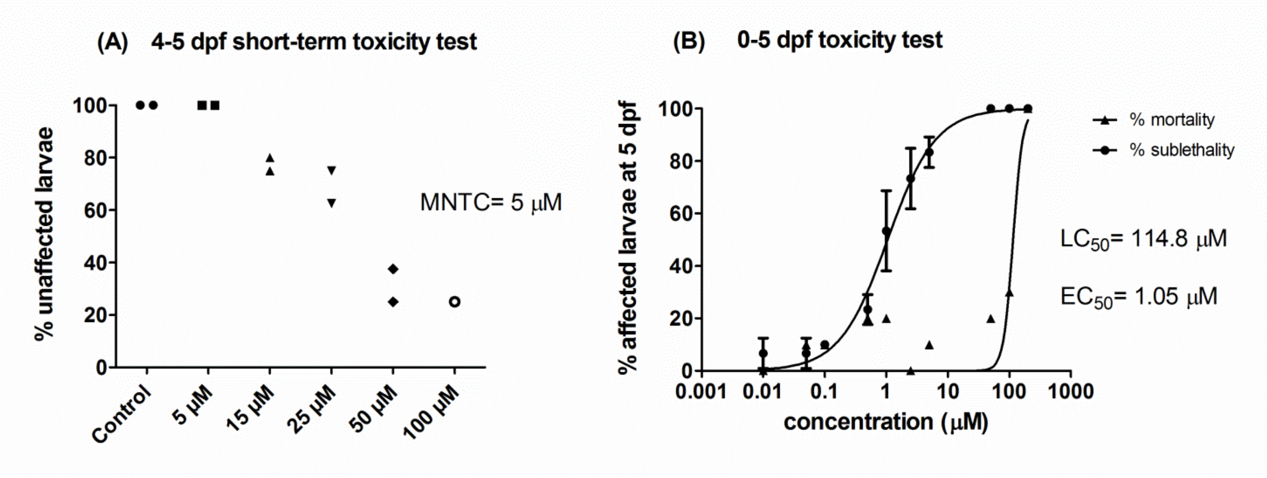

Figure S1: Toxic and non-toxic concentrations of mCPP in zebrafish embryos/larvae were determined from short-term larval (A) and zebrafish embryo toxicity tests (B). (A) Zebrafish larvae were exposed to mCPP from 4 days post fertilization (dpf) to 5 dpf in order to choose the concentrations for kinetics and behavioural studies. A maximum non-toxic concentration (MNTC), i.e. the applied concentration at which no significant effects were seen, was determined. Sub-lethal effects noted were posture imbalance and swimming abnormalities. Values are reported from two independent replicates, obtained from 10 larvae per concentration per replicate, except for 100 µM where only one experimental replicate was performed. (B) In order to assess the developmental effects of mCPP, zebrafish embryos were exposed from 0-5 dpf and the lethal (LC_{50}) and sub-lethal (EC_{50}) concentrations were calculated. LC_{50} was determined from an initial range-finding test conducted once with 10 embryos per concentration. Values for EC_{50} are obtained from 3 independent replicates and reported as mean ± SD from 10 larvae per concentration per replicate. Sub-lethal effects observed were heart edema, yolk-sac edema, decreased heart rate and absence of swim-bladder inflation.

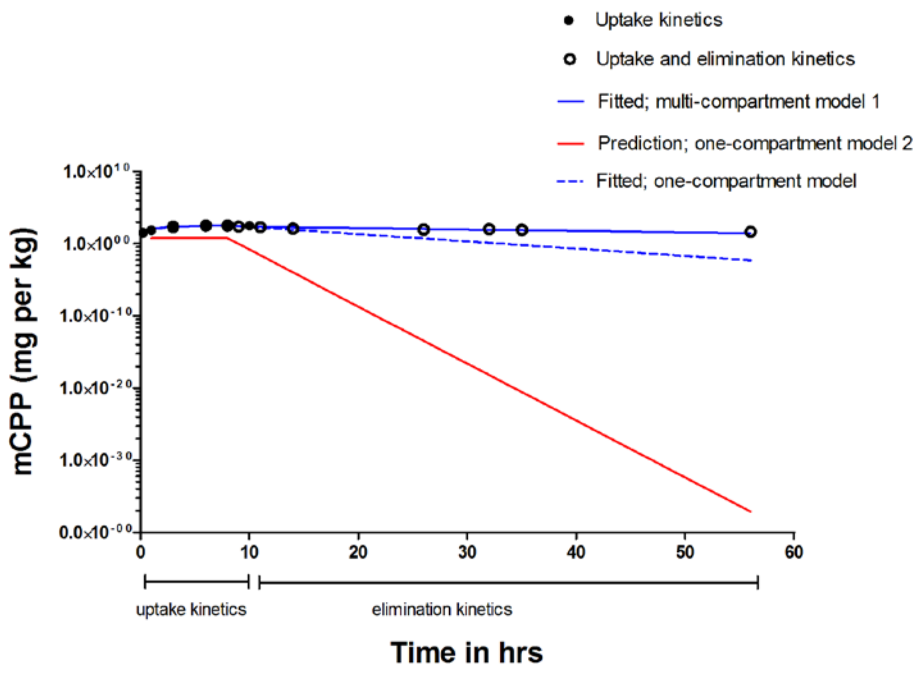

Figure S2: Semi-logarithmic plot of Figure 1, uptake and elimination kinetics of mCPP in zebrafish larvae. Filled circles show data from two independent experimental replicates focussing on uptake kinetics; open circles show data from two additional independent experimental replicates focussing on both uptake and elimination kinetics. Dashed line in blue shows the fitted one-compartment model 1; solid line in red shows the prediction of uptake and elimination based on one-compartment toxicokinetic model 2; solid line in blue shows the fitted multi-compartment toxicokinetic model 3.

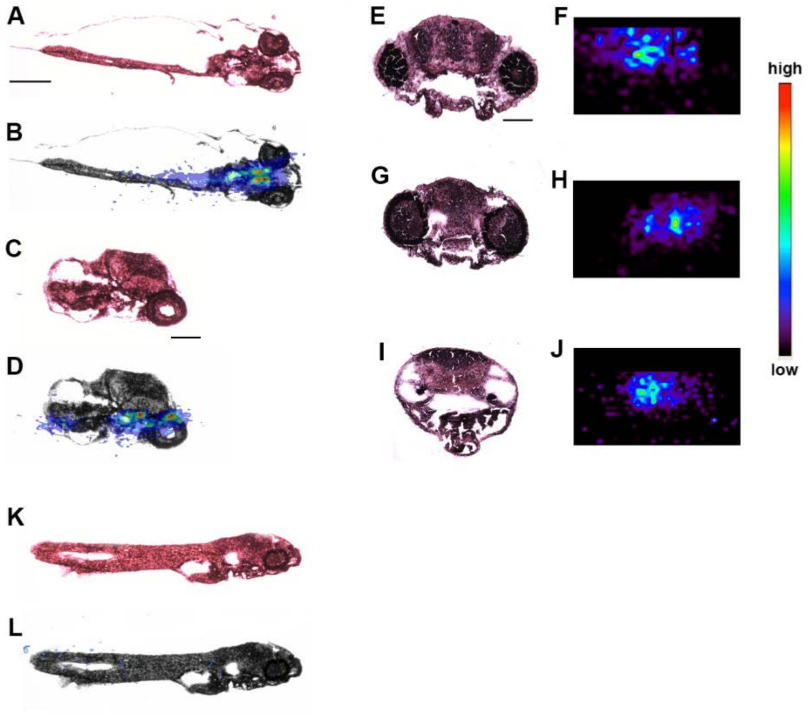

Figure S3: mCPP distribution in zebrafish larvae at 5 dpf exposed to 5 µM mCPP for 8 h, visualized by MALDI MSI. Optical images were obtained by Hematoxylin & Eosin staining. Coronal sections are depicted as optical image (A) and overlay of this optical image with the MALDI image (B), indicating mCPP distribution in the trunk region and with high intensity in the head region, scale bar: 300 µm. Sagittal sections with optical image (C) and overlay of the optical image with the MALDI image (D) show the accumulation of mCPP in the brain and the eye, scale bar: 150 µm. Transverse sections made across the head region through the forebrain (optical image - E and overlay with the MALDI image - F), midbrain (optical image - G, overlay with the MALDI image - H) and hindbrain (optical image - I, overlay with the MALDI image - J) confirm the presence of mCPP in the brain, scale bar: 100 µm. Sagittal sections with optical image of the unexposed fish (K) and overlay of an optical image with the MALDI image (L) show no mCPP signal.

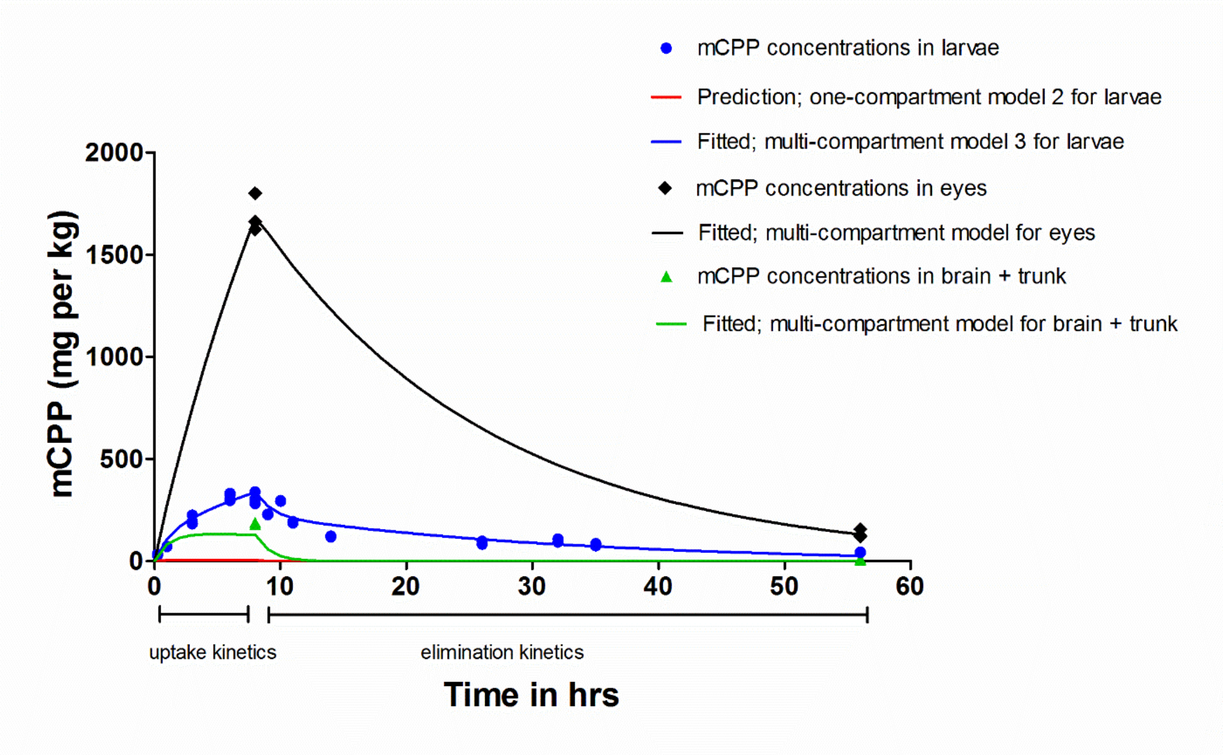

Figure S4: Uptake and elimination kinetics of mCPP in whole-body homogenates and dissected eyes, brain and trunk when eyes are separated from brain and trunk compartments. Larvae (16 per treatment) were exposed to 5 µM of mCPP at 5 dpf for 8 h and thereafter placed in mCPP-free medium for up to 48 h of elimination. A set of untreated larvae served as control. mCPP concentrations in larval whole-body homogenates and dissected eyes, brain and trunk were analysed by LC-MS/MS. In blue - uptake and elimination kinetics for whole larvae; in green - fitted multi-compartment model for brain and trunk; in black - fitted multi-compartment model for the eyes; in red - prediction based on the one-compartment logK_{ow} of mCPP. Data originate from two independent experiments (each data point was obtained from whole-body homogenates or tissues pooled from 16 larvae per time point per experiment).

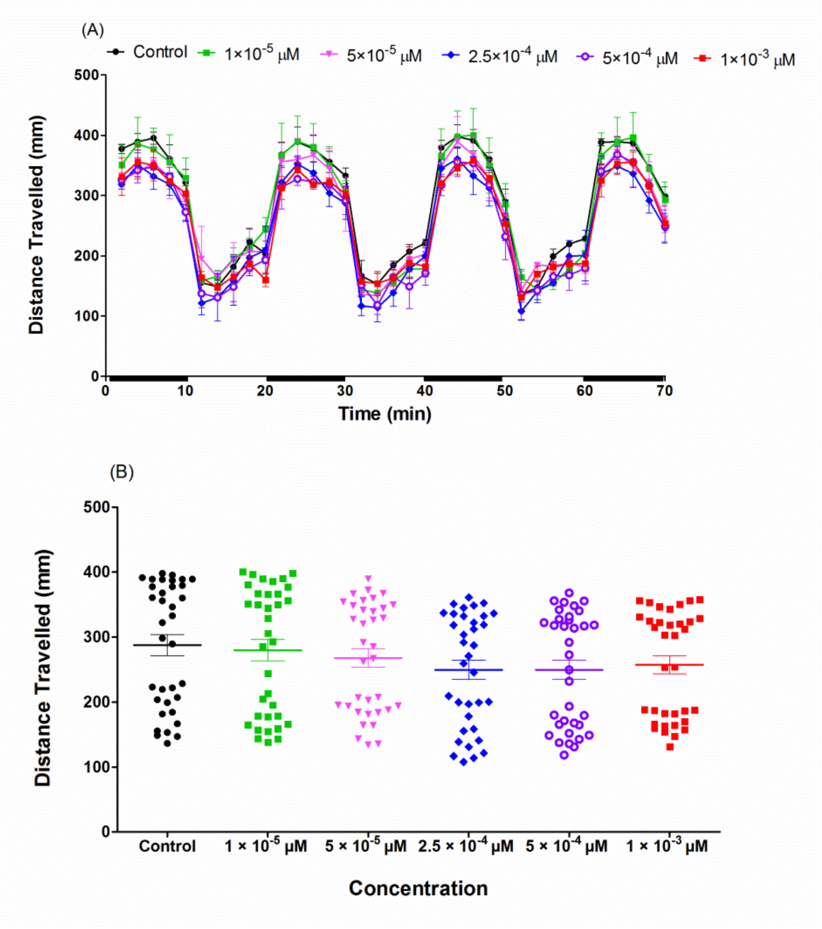

Figure S5: Locomotor activity of zebrafish larvae immediately after treatment with low concentrations of mCPP. Larvae at 5 dpf were exposed to the low concentration range of mCPP (1 × 10^{-5} µM – 1 × 10^{-3} µM). Distance travelled by the fish was analysed every 2 min for 70 min with dark (black bars on X-axis) and light phases alternating every 10 min (A) and distance travelled was plotted against different concentrations of mCPP (B). Data were analysed using repeated measures ANOVA. Data segregated and analysed for the effect of concentration at different lighting conditions showed no significant effect of low concentrations of mCPP on the locomotor activity of the zebrafish larvae. Values are reported as mean from three independent experiments; i.e., 3 independently prepared plates (n=8 larvae/concentration or control/plate) ± SEM.

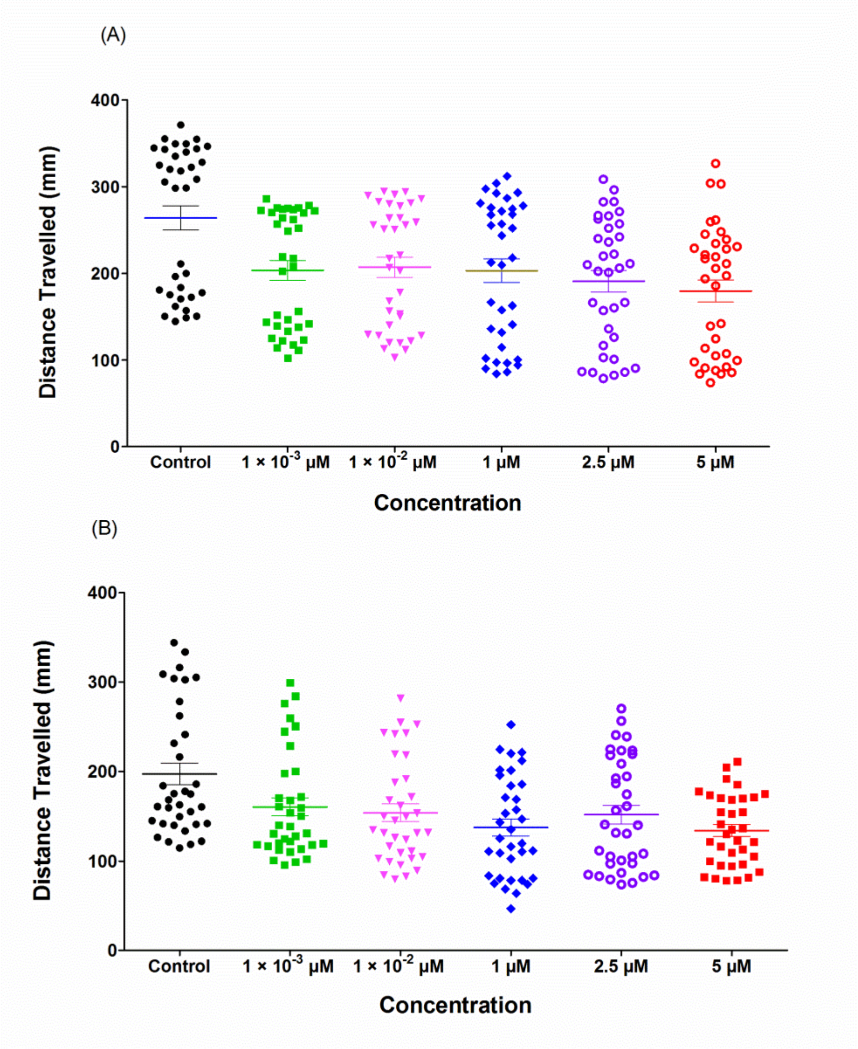

Figure S6: Effect of mCPP on the locomotor activity of zebrafish larvae. Larvae of 5 dpf were exposed to different concentrations of mCPP and tracked either immediately (A) or after 8 h of exposure (B). Distance travelled by the fish was plotted against different concentrations of mCPP. Data were assessed using repeated measures ANOVA. Values are reported as mean ± SEM from 3 independent experiments (n= 8 larvae/concentration or control/experiment).

Predicted one-compartment model 2 (developed by Hendriks et al. [1])

Table S1. Abbreviations and symbols used to describe the predicted one-compartment model 2

Abbreviation/ symbol	Units	Value	Description
w_w	kg	model input	body wet weight
K_{ow}	—	model input	octanol-water partition coefficient
C_w	$\mu g \cdot L^{-1}$	model input	chemical concentration in water
lipid	kg	model input	lipid weight
i	—	2 (for animals)	trophic level
j	—	0 - from water	type of chemical uptake
κ	—	0.25	rate exponent
$\rho_{CH2,i}$	$d \cdot kg^{-\kappa}$	68 (for animals)	lipid layer permeation resistance
$\rho_{H2O,j}$	$d \cdot kg^{-\kappa}$	$2.8 \cdot 10^{-3}$	water layer diffusion resistance
γ_0	$kg^{-\kappa} \cdot d^{-1}$	200 water breathing	water absorption-excretion coefficient
γ_1	$kg^{-\kappa} \cdot d^{-1}$	0.0006	biomass (re)production coefficient
$q_{T:c}$	$kg \cdot kg^{-1}$	1 (cold-blooded)	temperature correction factor
f_lipid	—	Equation S1	lipid fraction
k_{in}	$L \cdot kg^{-1} \cdot d^{-1}$	Equation S2	substance absorption rate constant
k_{out}	$kg \cdot kg^{-1} \cdot d^{-1}$	Equation S3	substance excretion rate constant
k_G	d^{-1}	Equation S4	growth dilution rate constant
C_{int}	$\mu g \cdot g^{-1}$	Equation S5	chemical internal concentration

- Fish lipid fraction in fish

$$f\_lipid = \frac{lipid}{w\_w} \qquad \textbf{(eq. S1)}$$

- Substance absorption rate constant

$$k_{in} = \frac{w\_w^{-\kappa}}{\rho_{H2O,j} + \frac{\rho_{CH2,i}}{K_{ow}} + \frac{1}{\gamma_0}}, \quad \frac{L}{kg \cdot d} \qquad \textbf{(eq. S2)}$$

- Substance excretion rate constant

$$k_{out} = \frac{1}{f\_lipid \cdot (K_{ow} - 1) + 1} \cdot k_{in}, \quad \frac{kg}{kg \cdot d} \qquad \textbf{(eq. S3)}$$

- Growth dilution rate constant

$$k_G = q_{T:c} \cdot \gamma_1 \cdot w\_w^{-\kappa}, \quad \frac{kg}{kg \cdot d} \qquad \textbf{(eq. S4)}$$

- Chemical internal concentration

$$\frac{d}{dt} C_{int}(t) = \frac{k_{in}}{1000} \cdot C_w(t) - (k_{out} + k_G) \cdot C int(t), \quad \frac{\mu g}{g \cdot d} \qquad \textbf{(eq. S5)}$$

Model runs:

Computational models were run in **ModelMaker 4** with the following settings:

a) Run

- start value: 0,
- stop value: last day of exposure,
- repeated run: no.

b) Integration

- random seed: 1,
- integration method: Runge-Kutta,
- output points: user defined (dependent on stop value)
- fixed step: no,
- accuracy: 10^{-6},
- minimum value: 10^{-10},
- approx. no of steps: 100,
- error scaling: a constant value (10).

c) Calibration

- Optimization run:
    - Method: Marquardt,
    - Weighting: ordinary least squares.
- Optimization Settings:
    - Convergence Change: 0.1,
    - Convergence Steps: 50,
    - Retry Count: 50,
- Marquardt settings:
    - Initial Lambda: 100,
    - Minimum Change: 1e-200,
    - Fractional Change: 0.01

Equation S6: From the 4-5 dpf short-term toxicity test on the larvae, maximum non-toxic concentration (MNTC), i.e. the applied concentration at which no significant effects were seen, was obtained by monitoring the abnormalities. From the MNTC, maximum internal non-toxic concentration (INTC) was calculated to compare to the toxicity of cocaine in zebrafish larvae [2]

$$INTC\ \left(\frac{mmol}{kg}\right) = MNTC\ (mmol.l^{-1}).\frac{k_{in}(l.kg^{-1}.h^{-1}}{k_{out}\ (h^{-1})})$$

INTC- maximum internal non-toxic concentration

MNTC- maximum non-toxic concentration

k_{in}- uptake rate constant

k_{out}- elimination rate constant

Equation S7: Based on the data obtained from the 0-5 dpf acute toxicity test, internal lethal and sub-lethal concentrations were calculated to be able to compare the effects of mCPP to those of cocaine on the development of the embryos:

$$ILC_{50}\ \left(\frac{mmol}{kg}\right) = LC_{50}\ (mmol.l^{-1}).\frac{k_{in}(l.kg^{-1}.h^{-1}}{k_{out}\ (h^{-1})})$$

$$IEC_{50}\ \left(\frac{mmol}{kg}\right) = EC_{50}\ (mmol.l^{-1}).\frac{k_{in}(l.kg^{-1}.h^{-1}}{k_{out}\ (h^{-1})})$$

ILC_{50}- internal lethal concentration

IEC_{50}- internal effective concentration

k_{in}- uptake rate constant

k_{out}- elimination rate constant

Table S2: Wet weight determination of the whole larvae and dissected tissues

(A)

No. of tubes	Weight of one fish (mg)	Average (mg)	Average (µg)	SD
**5dpf**				
1	0.37	0.37	368.25	15.61
2	0.37			
3	0.38			
4	0.39			
5	0.34			
**6dpf**				
1	0.34	0.34	335.63	48.87
2	0.27			
3	0.39			
**7dpf**				
1	0.34	0.37	373.54	28.22
2	0.37			
3	0.41			

Larvae at different stages were wet weighed in a 1.5 ml Eppendorf tube by gently aspirating the liquid medium before weighing. Weight of a larva was determined from each tube and an average weight of 360 ± 16 µg was obtained from 5-7 dpf.

(B)

No.of sets	Tissue	Weight per tissue (mg)	Weight per tissue (µg)	Average from three sets (µg)
Set 1	Brain	0.092	92	91.2
Set 1	Eyes	0.0651	65.1	68.8
Set 1	Trunk	0.1881	188.1	183.2
Set 2	Brain	0.0905	90.5	
Set 2	Eyes	0.07	70	
Set 2	Trunk	0.1792	179.2	
Set 3	Brain	0.0912	91.2	
Set 3	Eyes	0.07125	71.25	
Set 3	Trunk	0.1822	182.2	

Larval tissues were dissected and wet weighed in a 1.5 ml Eppendorf tube by gently aspirating the liquid medium before weighing. The average weight obtained from three independent measurements was used for the internal concentration measurements.

Table S3: Biotransformation

	Phase I % relative oxidation*		Phase II % relative glucuronidation*	
**Time in h**	**Experiment 1**	**Experiment 2**	**Experiment 1**	**Experiment 2**
0.25	1.8	1.8	1.3	1.3
1	1.5	1.2	1.1	0.8
3	1.6	1.6	1.1	1.1
6	2.0	1.9	1.4	1.4
8	1.7	1.8	1.2	1.3
10	2.0	2.0	1.4	1.5

* Metabolites were measured but absolute quantification could not be carried out because of the unavailability of standards. Therefore % relative oxidation and glucuronidation were calculated based on the ratio of the area counts of the metabolites mCPP-OH and mCPP-glucuronides to the area counts of the parent compound, mCPP, over time.

Table S4: Statistical analysis of the effect of mCPP on zebrafish larvae locomotion

Condition of exposure and comparisons	Concentration	*p* value	
		Light	Dark
Acute exposure, low concentrations compared to control	1×10^{-5} µM	0.594	0.206
	5×10^{-5} µM	0.314	0.0164
	2.5×10^{-4} µM	0.096	0.0329
	5×10^{-4} µM	0.275	0.202
	1×10^{-3} µM	0.383	0.289
Acute exposure, comparison of one concentration to the other	1×10^{-5} vs 5×10^{-5} µM	0.684	0.357
	1×10^{-5} vs 2.5×10^{-4} µM	0.319	0.432
	1×10^{-5} vs 5×10^{-4} µM	0.641	0.979
	1×10^{-5} vs 1×10^{-3} µM	0.732	0.817
	5×10^{-5} vs 2.5×10^{-4} µM	0.525	0.923
	5×10^{-5} vs 5×10^{-4} µM	0.959	0.333
	5×10^{-5} vs 1×10^{-3} µM	0.982	0.233
	2.5×10^{-4} vs 5×10^{-4} µM	0.542	0.408
	2.5×10^{-4} vs 1×10^{-3} µM	0.568	0.300
	5×10^{-4} vs 1×10^{-3} µM	0.945	0.834
Acute exposure, concentrations compared to control	1×10^{-3} µM	0.0126	0.000171**
	0.01 µM	0.0513	0.0006**
	1 µM	0.0024*	0.0033*
	2.5 µM	0.001*	3.75e-05**
	5 µM	0.000159**	9.28e-06**
Acute exposure, comparison of one concentration to the other	1×10^{-3} vs 1×10^{-2} µM	0.819	0.831
	1×10^{-3} vs 1 µM	0.313	0.595
	1×10^{-3} vs 2.5 µM	0.227	0.475
	1×10^{-3} vs 5 µM	0.044	0.207
	1×10^{-2} vs 1 µM	0.307	0.746
	1×10^{-2} vs 2.5 µM	0.245	0.389
	1×10^{-2} vs 5 µM	0.072	0.172
	1 vs 2.5 µM	0.931	0.271
	1 vs 5 µM	0.399	0.123
	2.5 vs 5 µM	0.405	0.585
8 h exposure, all concentrations compared to control	1×10^{-3} µM	0.413	0.00038**
	1×10^{-2} µM	0.090	0.002*
	1 µM	0.0049	0.00066**
	2.5 µM	0.0005*	0.0156
	5 µM	0.005	8e-05**
8 h exposure, comparison of one concentration to the other	1×10^{-3} vs 1×10^{-2} µM	0.183	0.913
	1×10^{-3} vs 1 µM	0.008	0.616
	1×10^{-3} vs 2.5 µM	0.0003**	0.303
	1×10^{-3} vs 5 µM	0.006	0.145
	1×10^{-2} vs 1 µM	0.178	0.601
	1×10^{-2} vs 2.5 µM	0.129	0.437
	1×10^{-2} vs 5 µM	0.435	0.171
	1 vs 2.5 µM	0.871	0.197
	1 vs 5 µM	0.410	0.384
	2.5 vs 5 µM	0.372	0.037

'α' value was set to 0.003 following Bonferroni's post-hoc analysis. *$p<0.003$.

References

[1] A.J. Hendriks, A. van der Linde, G. Cornelissen, D. Sijm, The power of size. 1. Rate constants and equilibrium ratios for accumulation of organic substances related to octanol-water partition ratio and species weight, Environ. Toxicol. Chem. 20(7) (2001) 1399-1420.

[2] K.T. Kirla, K.J. Groh, A.E. Steuer, M. Poetzsch, R.K. Banote, J. Stadnicka-Michalak, R.I. Eggen, K. Schirmer, T. Kraemer, Zebrafish Larvae Are Insensitive to Stimulation by Cocaine: Importance of Exposure Route and Toxicokinetics, Toxicological sciences : an official journal of the Society of Toxicology 154(1) (2016) 183-193.
